# Blood-borne virus testing in European emergency departments: current evidence and service considerations

**DOI:** 10.1093/eurpub/ckaf103

**Published:** 2025-07-04

**Authors:** Elizabeth Smout, Murad Ruf, Maria Buti, Inês Vaz Pinto, Gaia Nebbia, Laura Hunter, Mark A Aldersley, Catarina Esteves, Diogo Medina, Jordi Llaneras, Sam Douthwaite, Emma E Page

**Affiliations:** Public Health, Gloucestershire NHS Trust, United Kingdom; Public Health, Medical Affairs, Gilead Sciences Europe, London, United Kingdom; Liver Unit, Hospital Universitari Vall d’Hebron, Barcelona, Spain; HIV Unit, Cascais Hospital, Cascais, Portugal; Department of Infection, Guy’s and St Thomas’ NHS Foundation Trust, London, United Kingdom; Department of Emergency Medicine, Guy’s and St Thomas’ NHS Foundation Trust, London, United Kingdom; Liver Unit, Leeds Teaching Hospitals NHS Trust, Leeds, United Kingdom; HIV Unit, Cascais Hospital, Cascais, Portugal; FOCUS Program, Gilead Sciences, Madrid, Spain; Emergency Department, Hospital Universitari Vall d’Hebron, Barcelona, Spain; Department of Infection, Guy’s and St Thomas’ NHS Foundation Trust, London, United Kingdom; Department of Virology, Leeds Teaching Hospitals NHS Trust, Leeds General Infirmary, Leeds, United Kingdom

## Abstract

Innovative testing approaches are needed to meet global targets for the blood-borne viruses (BBVs) HIV, hepatitis B virus (HBV) and hepatitis C virus (HCV). We conducted a systematic review of BBV testing in emergency departments (EDs) in Europe to evaluate prevalence, effectiveness of ED testing and linkage to care (LTC). We searched PubMed, Embase and Cochrane Library for articles on ED BBV testing published between January 2012 and July 2022. Studies conducted outside Europe or prior to 2012 were excluded owing to epidemiological and healthcare service variation, together with studies that did not report core parameters. Reference lists from included articles were manually searched. Seventeen original articles met the inclusion criteria. Seven studies reported on HIV testing only. ED prevalence: HIV Ab, 0.0%–1.1%; HBsAg, 0.2%–0.9%; and HCV RNA, 0.2%–3.9%. BBV testing uptake varied by policy and offer methodology: opt-out, provider-initiated: 9.7%–44.2%; electronic health record (EHR) modification: 52.1%–88.9%; and opt-in, provider-initiated: 3.9%–37.7%. LTC rates were 8.1%–100% and varied by BBV, generally highest for HIV and lowest for HCV. There was variable detail in outcome reporting and description of clinical LTC pathways. ED BBV testing in Europe is feasible and identifies high numbers of infections (including, where reported, new diagnoses and disengaged patients), often among marginalized populations who use open-access EDs for healthcare. Factors associated with higher levels of sustained testing uptake included opt-out testing (vs opt-in), EHR (vs provider-initiated) and integration of community services. We propose a toolkit of components necessary for a high-performing ED BBV testing programme.

## Introduction

Blood-borne viruses (BBVs), including human immunodeficiency virus (HIV), hepatitis C virus (HCV) and hepatitis B virus (HBV), are major public health threats, associated with significant morbidity and mortality globally [1–3].

Global elimination targets exist for all three BBVs. The World Health Organization (WHO) proposed global targets for eliminating viral hepatitis as a major public health problem by 2030 [4].

BBVs disproportionately affect marginalized populations [1, 5, 6], including people who inject drugs, men who have sex with men, transgender people, sex workers, migrants, homeless people and prison inmates [1, 5–9]. These groups often have poor access to primary care, instead accessing the healthcare system through open-access hospital emergency departments (EDs) [10, 11].

BBV testing in the ED has the potential to identify patients with undiagnosed BBV infection and those who have disengaged with care [1]. Opportunistic, universal ED screening therefore has value as a ‘safety net’ [12]. While the UK National Institute for Health and Care Excellence (NICE) and the British HIV Association recommend HIV testing in the ED in high-prevalence areas (≥0.2%) [13], NICE does not yet recommend the same for HBV and HCV. The European Centre for Disease Prevention and Control (ECDC) recommends systematic BBV screening in EDs in high-prevalence areas (≥5% for HBV and HCV; ≥1% for HIV) [14].

A previous systematic review of BBV testing within EDs concluded that systematic BBV testing in EDs is feasible and acceptable but that linkage to care (LTC) needed to be improved [1]. However, many studies were from the first decade of the 21st century and included the USA, so cannot be extrapolated to European EDs due to differences in epidemiology, healthcare systems and evolution of testing approaches over time. Therefore, we performed an updated systematic review of recent studies in Europe only, accompanied by an opinion piece on lessons learned from its implementation.

## Methods

We conducted a systematic review of BBV testing in European EDs between January 2012 and July 2022. The review evaluated prevalence of diagnosed active BBV infection among people attending EDs, feasibility and acceptability of ED testing, type of testing (targeted, opt-in, opt-out), testing methodologies (provider-led vs using electronic health record [EHR] modification), LTC and cost-effectiveness.

### Search inclusion and exclusion criteria

The Population, Intervention, Comparison, Outcome and Study design (PICOS) framework for this systematic review was as follows:

- Population: ED attendees
- Intervention: BBV testing within EDs and LTC
- Comparison: none
- Outcome: BBV test offer, uptake, seroprevalence, test positivity, LTC, retention in care
- Study design: observational studies, retrospective and prospective cohort studies, qualitative studies

Test uptake was considered to be an indicative function of acceptability and real-world feasibility. LTC referred to people diagnosed with BBVs engaging with care following diagnosis. Only studies in English were included. Publications from outside Europe or published before 2012 were excluded.

### Search strategy

We searched PubMed, Embase and the Cochrane Library in July 2022. An initial search was carried out using terms listed in Table 1.

**Table 1.** List of search terms used.

Search term
(HIV) AND (Opt-out) AND (Europe)
((HIV) OR (human immunodeficiency virus) OR (HBV) OR (hepatitis b) OR (HCV) OR (hepatitis C)) AND ((emergency department) OR (ED)) AND (opt out)
((HIV) OR (human immunodeficiency virus) OR (HIV-1) OR (hepatitis) OR (HCV) OR (HBV) OR (hepatitis B) OR (hepatitis C)) AND ((blood borne virus) OR (BBV) OR (bloodborne virus) OR (blood borne))
((HIV[Title]) OR (HCV[Title]) OR (HBV[Title]) OR (human immunodeficiency virus[Title]) OR (hepatitis c[Title]) OR (hepatitis c[Title])) AND ((emergency department*[Title]) OR (ED[Title]))

Abbreviations: BBV, blood-borne virus; ED, emergency department; HBV, hepatitis B virus; HCV, hepatitis C virus; HIV, human immunodeficiency virus.

Title, abstract and full text of publications were assessed. Additional publications were identified through manual reference list searches of all articles retrieved.

### Data extraction

**Figure 1.**
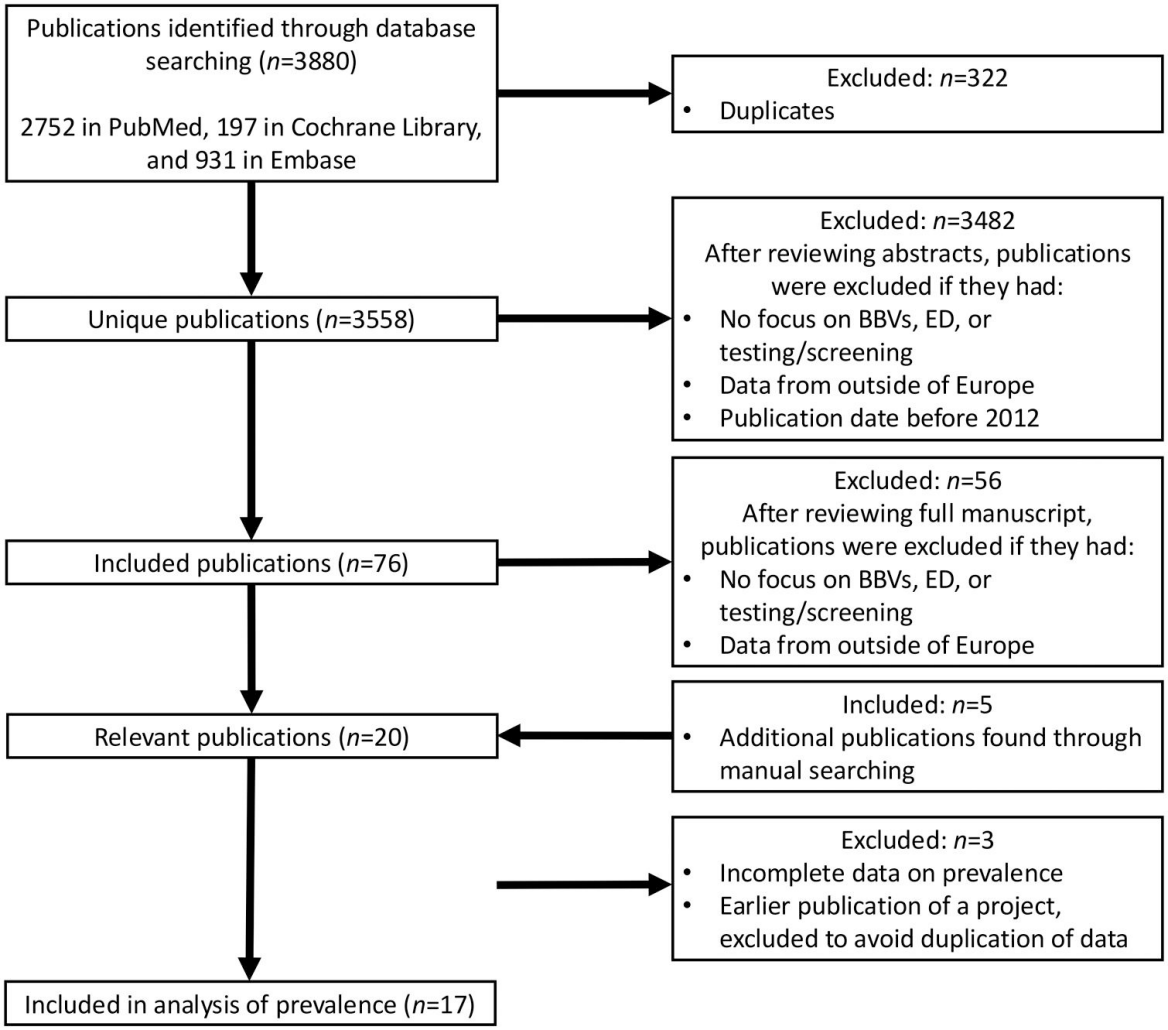
Flow diagram of publications included in the systematic review. Abbreviations: BBV, blood-borne virus; ED, emergency department.

Publications identified by the initial search strategy were imported into Mendeley Reference Manager and any duplicates removed. All titles were reviewed by authors E.S. and M.R.; studies investigating settings other than the ED and publications including non-European data were removed. The full texts were then examined for eligibility criteria (Fig. 1). Authors were contacted to request the full text if these were not available.

## Results

In total, 3880 papers were identified across all searches. Of these, 322 were duplicates. After reviewing, there were 20 relevant publications, including two papers provided by the authors, published after the original search and three papers found through manual reference list searching.

### Study validity assessment

There were significant variations in methodology and local populations. Therefore, meta-analysis (including risk of bias) was considered unsuitable. Most studies included were also included in a previous systematic review and were categorized as seriously or critically biased [1]. Factors included insufficient sample size, study duration, number of centres and variation in testing and LTC methodologies. Reporting of results was also not standardized. Furthermore, none of the studies had an associated Consolidated Standards of Reporting Trials (CONSORT) statement or good practice protocol.

### Systematic review findings

In total, 17 studies provided data on prevalence on one or more BBV infections in adult patients only. Fifteen studies were included in the analysis set for HIV testing. Nine studies were included for HBV testing. Nine studies were also included for HCV testing (O’Connell *et al.* [15] was excluded for HCV as HCV RNA prevalence data were not reported). Three publications of the 20 relevant publications were excluded. Phyu *et al.* [16] was a real-world extension of the Get Tested Leeds project (Smout *et al.* [12]); therefore, the paper by Smout *et al.* was excluded from this analysis to avoid duplication. Two articles were omitted as they reported incomplete prevalence data [17, 18].

**Figure 2.**
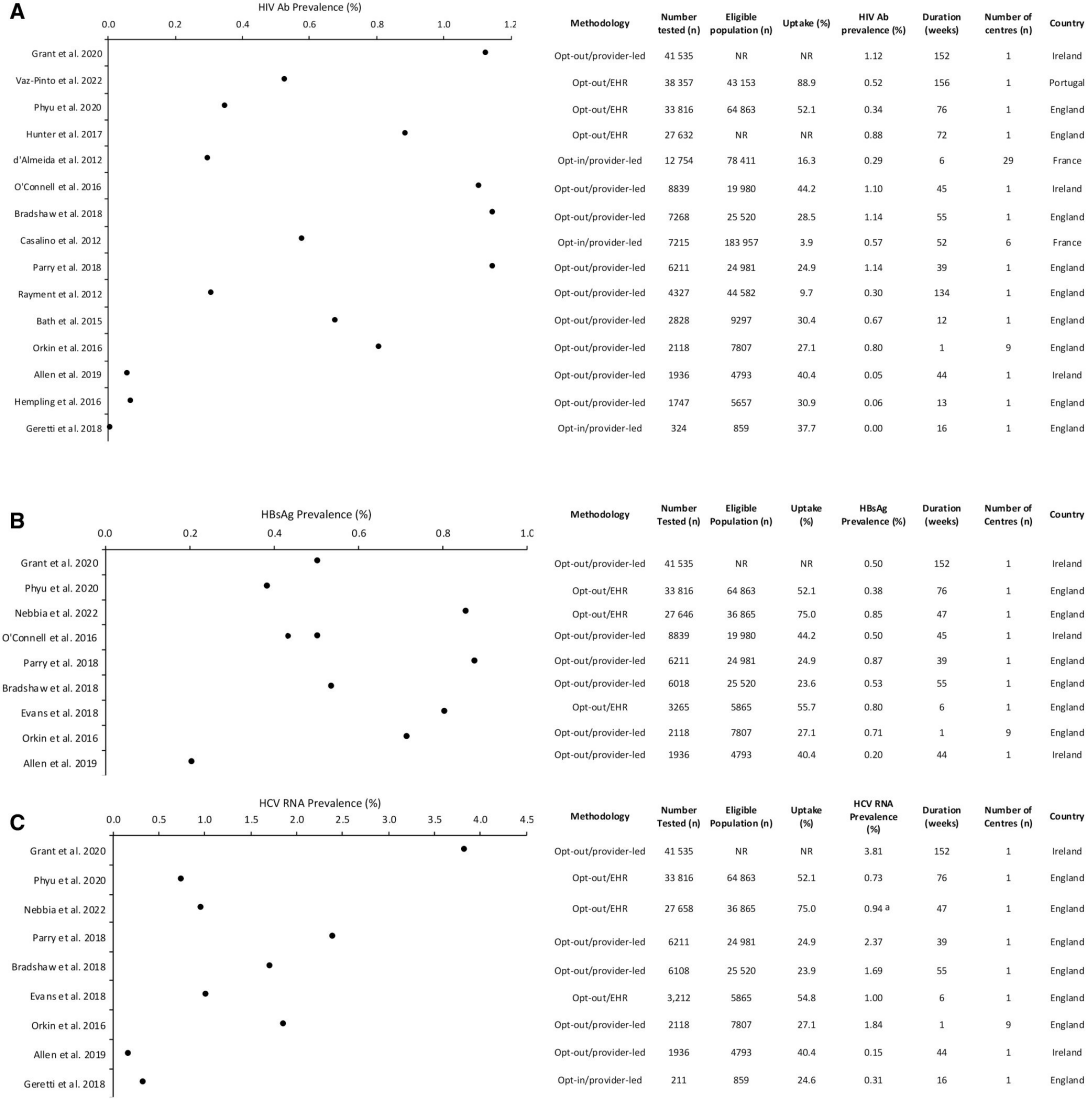
Screen-detected prevalence among ED attendees, ordered by number tested, of (A) HIV Ab, (B) HBsAg and (C) HCV RNA. (A) Nebbia *et al*. used HCV cAg. (B) Palfreeman *et al*. (2013) and Luiken *et al*. (2017) are not included in this figure as they did not report prevalence data. (C) O’Connell *et al*. (2016) is not included as the data for HCV RNA prevalence was unclear with respect to the total HCV prevalence data. Abbreviations: Ab, antibody; cAg, core antigen; ED, emergency department; EHR, electronic health record; HBsAg, hepatitis B surface antigen; HCV, hepatitis C virus; HIV, human immunodeficiency virus; NR, not reported.

Eleven of the studies included were from England [16, 19–28], with the remaining six from Ireland, France and Portugal [3, 15, 29–32]. Seven studies examined testing for all three BBV infections, seven studies considered HIV only, two included HBV and HCV only and one study looked at HIV and HCV only. Across all three BBV infections, the eligible population ranged from 859 to 183 957 patients (Fig. 2). Of these, the number of patients tested ranged from 113 to 41 535. Testing uptake ranged from 3.9% to 88.9% and varied by testing methodology (Fig. 2).

### BBV testing uptake and prevalence of screening

In provider-led methodologies, testing was requested by the healthcare professional (HCP) attending the patient. In methodologies using EHR modification, the EHR system was programmed to pre-select BBV tests for any blood test orders in the ED.

#### Human immunodeficiency virus

Reported HIV antibody (Ab) prevalence ranged from 0.0% to 1.1% (Fig. 2A). Of the studies including HIV testing, the majority (*n* = 9) used an opt-out/provider-led approach [3, 15, 21–27, 29], three studies used an opt-in/provider-led approach [28, 30, 31] and three studies used an opt-out/EHR approach [16, 26, 32]. For the studies that used opt-out/EHR, testing uptake ranged from 52.1% to 88.9% (Fig. 2A), while the opt-out/provider-led method had an uptake of 9.7–44.2% and the opt-in/provider-led method had an uptake of 3.9–37.7%. Definition of LTC varied among studies (e.g. referral- or treatment-initiated). Of the total number of patients diagnosed, LTC ranged from 8.1% to 100% [15, 16, 21, 23, 27, 29, 30, 33].

#### Hepatitis B virus

The prevalence of hepatitis B surface antigen (HBsAg) ranged from 0.2% to 0.9% (Fig. 2B). Of the nine studies including HBV testing, all involved opt-out approaches; six were provider-led [3, 15, 21–23, 29] and three used EHRs to identify patients [16, 19, 20]. Studies using opt-out/EHRs reported an uptake of 52.1–75.0%, while studies using the opt-out/provider-led approach reported an uptake of 23.6–44.2% (Fig. 2B). Of the total number of patients diagnosed, LTC ranged from 27.7% to 95.5% [15, 16, 19–21, 23, 29].

#### Hepatitis C virus

The prevalence of active HCV infection (including one study [19] reporting HCV core antigen rather than RNA) ranged from 0.2% to 3.9% (Fig. 2C). Of the nine studies including HCV testing, five used an opt-out/provider-led approach [3, 21–23, 29], one used an opt-in/provider-led approach [28] and three used an opt-out/EHR approach [16, 19, 20]. Studies using opt-out/EHR approaches reported an uptake of 52.1–75.0% [16, 19, 20], while studies with an opt-out/provider-led approach reported uptake ranging from 23.9% to 40.4%. Uptake was below 28% in three of the five studies (Fig. 2C) [3, 21–23, 29]. For the study with an opt-in/provider-led approach, percentage uptake was 24.6% [28]. Of the total number of patients diagnosed, LTC ranged from 12.9% to 48.6% [16, 19–21, 23, 29].

## Discussion

### Which BBVs should be screened for in the ED?

This study outlines the significant emerging European evidence supporting ED screening for all three BBVs [34]. The predominance of HIV-focused studies is unsurprising; ED HIV testing has a longer history than testing for other BBV infections [1]. In the last decade, direct-acting antivirals for HCV treatment have become available, which likely spurred interest in systematic HCV testing.

### Key factors influencing optimization of screening uptake and LTC

This review confirms the finding by Simmons *et al.* that BBV screening in the ED is acceptable to patients, can be sustainable for services, leads to high uptake and detects high numbers of infections, including, where recorded, new infections and patients previously diagnosed but lost to follow-up [1]. Although data on new diagnoses vs patients ‘previously diagnosed but disengaged’ are not consistently reported, we believe there is emerging evidence that BBV testing identifies a substantial total number of patients requiring LTC.

While many earlier studies did not distinguish between new and known infections, the more recent ones did [12, 19, 33]. Where differential LTC was reported, LTC was generally more challenging for those who had previously disengaged from care. The reasons are not reported but are likely to include social factors and current treatment service configurations. We hope future qualitative research will shine a light on contributing factors and the need for patient-centric care models.

Emerging data from the English ED BBV opt-out program (Apr 2022 to Mar 2024) confirm that patients with previous diagnoses of HIV who disengaged with care were more difficult to re-engage than those with new diagnoses [35]. We recommend making the optimization of LTC pathways a research focus.

With appropriate LTC, BBV screening can result in significant numbers of patients engaging with specialist care and treatment [1, 19]. However, there is significant variation in outcomes along the patient pathway.

#### Universal opt-out testing may help to destigmatize BBV screening

The majority of studies identified were opt-out [3, 15, 16, 19–27, 29, 32], requiring minimum pre-test information to be provided to patients as testing becomes routine departmental policy. In the three studies using opt-in screening that reported uptake data, uptake ranged from 3.9% to 25.9% [28, 30, 31], compared with uptake ranging from 9.7% to 88.9% among opt-out studies [3, 15, 16, 19–27, 29, 32]. This large range is likely explained by differences in testing methodology, with EHR models generally achieving higher uptake than provider-led models (discussed below) [1].

Initiatives that use opportunistic opt-out screening would be expected to lead to higher uptake than opt-in testing [1]. Universal opt-out testing helps to normalize and destigmatize BBV screening and removes potential barriers that might result from HCPs being unwilling to discuss or offer BBV screening [1]. In contrast, targeted risk-based screening requires initiative from the HCP to identify at-risk patients. Furthermore, this approach requires patients to be both aware of and willing to disclose potentially stigmatizing BBV exposure.

#### EHR modification is effective and sustainable

Automating the testing request involves modifying the EHR system or electronic pathology order sets to opportunistically pre-select BBV tests when ordering routine blood tests. Figure 2A–C show that studies using EHR modification achieved high testing uptake (52.1–88.9%), sustained over several months [16, 19, 20, 26, 32]. In contrast, uptake was generally lower in programmes that used provider-led methodology [3, 15, 21–25, 27–31]. When multiple BBVs [3, 15, 16, 21–23, 29] were tested for simultaneously, testing uptake was generally equal across all three BBVs. However, one study found that concomitant HIV screening reduced uptake of HCV testing [28].

In the FOCUS-TEST model developed in the USA and deployed in Spain and Portugal, an EHR algorithm determines patient eligibility and also orders the test [34]. In comparison, UK models utilize a simpler EHR order set modification to link BBV tests to routine blood test orders [19].

Using an automated, integrated serology request overcomes barriers to testing by operationalizing and normalizing the process of universal screening for HCPs [32]. Removing the need for the HCP to individually order the test reduces the additional administrative burden and minimizes operational challenges of introducing screening in a busy ED. However, different EDs often have different EHR systems, thus requiring bespoke adaptation.

### Even in opt-out testing, patients provide informed consent

Even in opt-out screening, informed consent with the explicit option to decline testing is an important consideration: in most opt-out testing programmes reviewed, patients were made aware of the organization’s screening policy using multiple methods often in combination, including posters in the ED, multilingual leaflets provided to patients at registration and verbal information before a blood test [3, 15, 16, 18, 21, 22, 24, 25, 27, 30, 31].

Although WHO and ECDC recommend removing requirements for written consent for HIV and viral hepatitis testing [14], consent guidance for HIV testing in particular varies across European countries [14]. The UK is moving towards ‘implied consent’, the approach generally adopted for most routine blood tests in which consent is presumed through general consent to medical care [36]. By allowing the clinician to take blood, the patient effectively consents to the test despite no prior discussion of the implications of abnormal results [36]. This approach is being implemented in most London EDs and many other urban EDs around England participating in the current ED BBV opt-out testing program [37]. Consent legislation for HIV testing is generally stricter than that for viral hepatitis [36], likely for historical reasons as HIV infection was once untreatable and terminal. However, as highly effective antiviral therapy is now available for all three BBVs, this ‘HIV testing exceptionalism’ [36] may benefit from renewed public discussion and potential alignment.

### ‘No news is good news’

In the majority of the testing initiatives, only positive results are communicated to the patient (so the message is ‘no news is good news’). In the case of insufficient samples, indeterminate/equivocal tests or human error (in which the BBV sample was not drawn), there is a risk that the patient believes a BBV test is negative when it has not been performed. In the authors’ experience to date, insufficient samples have not been a problem, and indeterminate/equivocal tests are rare. In some centres, the concomitant biochemistry samples were tested in these cases. Indeterminate/equivocal results that remain are communicated to patients and they are encouraged to attend for repeat testing. Perhaps more common and more important than indeterminate/equivocal results are instances where the HCP taking the blood sample does not actually take the BBV sample and the patient thinks the test is negative, unaware that the BBV test was not performed. This scenario and potential solutions will need to be individually considered by local hospitals’ risk management committees, and they will need to consider whether the risk of this happening is outweighed by the benefits of the overall programme. We are unable to give a recommendation on this point.

### Dedicated LTC pathways are important but challenging

Only with robust LTC pathways will a testing intervention be ethical, have clinical and public health benefits and realize the health economic aspects of the intervention. Assessment of LTC rates needs to be a central part of any evaluation of effectiveness. However, with some exceptions, the majority of publications did not report LTC outcomes or clinical outcomes beyond linkage to specialist services and reported definitions of LTC were not uniform.

LTC can be challenging due to both service and patient factors [15, 16, 19–21, 23, 27, 29, 30, 32]. From a service perspective, LTC pathways for HBV and HCV infections are less well-established than those for HIV infection. Considering patient factors, LTC is particularly challenging for those diagnosed with HIV, especially for those individuals actively using intravenous drugs [15, 22, 23]. Emerging data from the English BBV ED opt-out program suggest that patients with previous diagnoses who disengaged with care are often more difficult to re-engage than those with new diagnoses [35].

#### Communication between stakeholders is necessary for effective LTC

Importantly, ED staff should only be responsible for testing, not LTC. Therefore, clear, locally agreed pathways need to exist between the testing laboratory and specialist clinical teams to ensure that positive results are reported and acted upon in a reliable and timely fashion [16].

Three recent projects that reported relatively high LTC included an LTC coordinator or project manager—a member of staff working in close collaboration with clinical teams responsible for conveying reactive test results to patients and encouraging them to engage with clinical services [19, 33]. Clear LTC pathways for patient notification and LTC via clinical teams or a care coordinator are important, minimizing the additional workload of frontline ED staff ordering the test, thus supporting ED clinical buy-in [19].

#### Integration with community services

Particularly for HCV infection, sharing information with community homelessness and drug services is important for finding and engaging with patients [19, 20, 23]. LTC networks need to embrace clinical teams and community-based teams and, where available, peer support workers should engage with patients, accompany them to clinic appointments and offer support throughout treatment [19]. Lessons from the more complex LTC pathways for HCV likely also apply to ED re-diagnosed patients with HIV infection who have disengaged from care.

### Is BBV screening in the ED cost-effective?

Screening for HIV is generally accepted as cost-effective in areas where undiagnosed prevalence is ≥0.1% [14, 18]. In 2018, ECDC suggested universal HBV and HCV testing when the overall prevalence is ≥2%, but highlighted the paucity of available evidence [14]. A 2022 economic analysis of data from two large, real-world, UK-based HBV and HCV ED testing projects found that testing remained cost-effective at ≥0.25% HBsAg prevalence and ≥0.49% HCV RNA prevalence [38]—much lower than the 2% thresholds suggested by ECDC [14]. Screen-diagnosed BBV prevalence in all our identified studies was well above these thresholds.

### A proposed ED screening and LTC pathway

**Figure 3.**
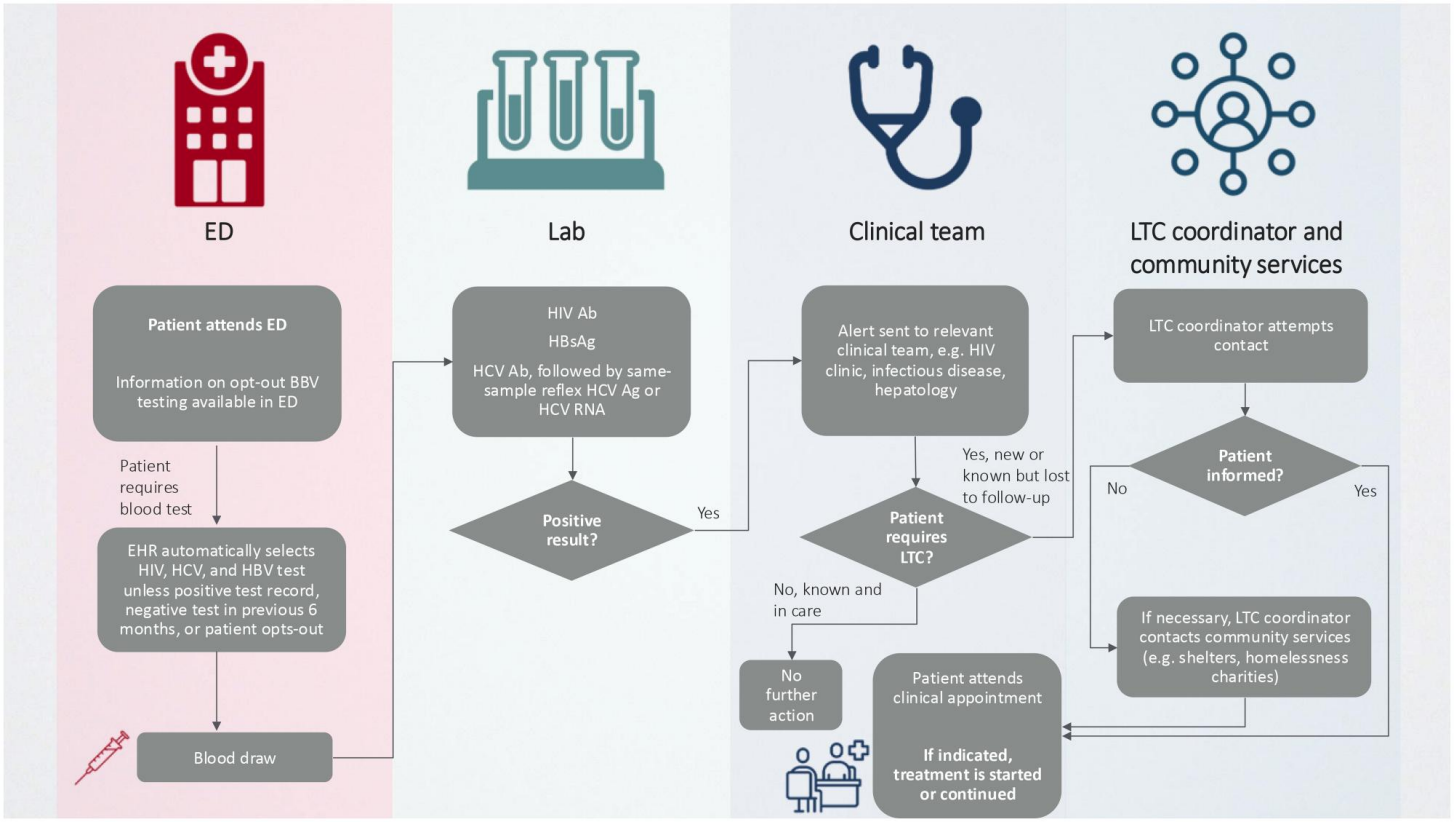
Suggested schematic ED screening and LTC pathway. Abbreviations: Ab, antibody; Ag, antigen; BBV, blood-borne virus; ED, emergency department; EHR, electronic health record; HBsAg, hepatitis B surface antigen; HBV, hepatitis B virus; HCV, hepatitis C virus; HIV, human immunodeficiency virus; LTC, linkage to care.

A key aim of this review is to provide a best practice ‘toolkit’ of processes and strategies to optimize uptake of ED-based BBV screening and successful LTC in Europe based on identified studies and co-authors’ real-world experience running such initiatives. These are summarized in Fig. 3; however, each ED will need to develop its own local protocols based on the department’s overall procedures and in collaboration with teams involved in each stage of the screening and LTC pathway.

Overall, our recommendation includes several important features:

- Formal ED operational group including key members involved along the patient pathway.
- Agreed screening policy: only positive results are communicated (the message to patients is ‘no news is good news’). Agreed local policy that ED staff perform the blood draw but are not responsible for communicating the test result.
- Opt-out testing through EHR or order set modification of all adult patients requiring a blood test in the ED, optionally limited to patients in high-prevalence age cohorts.
- Testing of HIV-Ab, HbsAg and HCV-Ab with same-sample reflex HCV-confirmatory testing.
- Broad local stakeholder engagement in LTC pathway including community services, particularly for HCV infection.

### Study limitations

The studies included in the systematic review are highly varied in their methods, with an absence of standardized reporting that is reflected in their outcomes. The included studies were carried out in England [18–28], France [30, 31], Ireland [3, 15, 29] and Portugal [32], with the majority (>60%) taking place in England, potentially because of the absence of clear ED screening guidelines in most European countries. Therefore, this systematic review has a bias towards findings in England. Nevertheless, the results of non-UK studies were generally consistent with those conducted in England, showing high burden of disease and feasibility of opt-out BBV testing [3, 12, 16, 19, 21, 23–32]. These results also concur with those from US studies [1]. The seroprevalence rates identified by ED screening in studies included in this review are substantially higher than estimated prevalence rates in the general population, supporting the notion that the ED is an appropriate setting for opportunistic BBV screening [14, 39, 40].

## Conclusions

The studies reviewed here demonstrate that BBV testing in European EDs is feasible and acceptable and detects high numbers of new infections, particularly in marginalized populations. With appropriate LTC models, testing is likely to be cost-effective. Opt-out testing, especially in combination with EHR modification, is associated with higher levels of sustained testing uptake than opt-in, provider-led testing. We propose a simple toolkit of evidence-based components necessary for a high-performing ED BBV testing programme. More empiric evidence on ED BBV testing and LTC from different European countries is necessary.

## Data Availability

All data analysed in the systematic review are available in the public domain. The datasets are available from the corresponding author on reasonable request. Key points Blood-borne viruses (BBVs) disproportionately affect marginalized populations that often have poor access to healthcare. Studies in this systematic review show that BBV testing in hospital emergency departments detects high numbers of new infections and, with appropriate linkage to care, is likely to be cost-effective in Europe. While there was considerable variation in testing approaches, opt-out testing, especially in combination with electronic health record modification, was consistently associated with higher levels of sustained testing uptake than traditional opt-in, provider-led testing. We discuss important components of a testing system and propose a schematic clinical pathway based on the available evidence and expert opinion. We recommend an informed multi-stakeholder dialogue and review to harmonize national testing consent recording requirements across Europe (including differences between HIV and viral hepatitis) in order to reduce persistent late diagnoses in the third decade of the 21st century.
